# Home-based virtual reality training after discharge from hospital-based stroke rehabilitation: a parallel randomized feasibility trial

**DOI:** 10.1186/s13063-019-3438-9

**Published:** 2019-06-07

**Authors:** Lisa Sheehy, Anne Taillon-Hobson, Heidi Sveistrup, Martin Bilodeau, Christine Yang, Vivian Welch, Alomgir Hossain, Hillel Finestone

**Affiliations:** 1 Bruyère Research Institute, 43 Bruyère St., Ottawa, ON K1N 5C8 Canada; 2 School of Rehabilitation Sciences, Faculty of Health Sciences, University of Ottawa, Guindon Hall, 451 Smyth Road, Ottawa, ON K1H 8M5 Canada; 3 Bruyère Continuing Care, 43 Bruyère St., Ottawa, ON K1N 5C8 Canada; 4 Division of Physical Medicine and Rehabilitation, Department of Medicine, Faculty of Medicine, University of Ottawa, 505 Smyth Rd., Ottawa, ON K1H 8M2 Canada; 5 School of Epidemiology and Public Health, Faculty of Medicine, University of Ottawa, 600 Peter Morand Cres., Ottawa, ON K1G 5Z3 Canada; 6 University of Ottawa Heart Institute, 40 Ruskin St., Ottawa, ON K1Y 4W7 Canada; 7 Institute for Clinical Evaluative Sciences (ICES), uOttawa, Ottawa Hospital, Civic Campus, 1053 Carling Ave., Box 684, Administrative Services Building, 1st Floor, Ottawa, ON K1Y 4E9 Canada; 8 Élisabeth Bruyère Hospital, 43 Bruyère St., Ottawa, ON K1N 5C8 Canada

**Keywords:** Virtual reality, Stroke, Telerehabilitation, Rehabilitation, Technology

## Abstract

**Background:**

Virtual reality training (VRT) uses computer software to track a user’s movements and allow him or her to interact with a game presented on a television screen. VRT is increasingly being used for the rehabilitation of arm function, balance and walking after stroke. Patients often require ongoing therapy post discharge from inpatient rehabilitation. Outpatient therapy may be limited or inaccessible due to waiting lists, transportation issues, distance etc.; therefore, home-based VRT could provide the required therapy in a more convenient and accessible setting. The objectives of this parallel randomized feasibility trial are to determine (1) the feasibility of using VRT in the home post stroke and (2) the feasibility of a battery of quantitative and qualitative outcome measures of stroke recovery.

**Methods:**

Forty patients who can stand for at least 2 min and are soon to be discharged from inpatient or outpatient rehabilitation post stroke are being recruited in Ottawa, Canada and being randomized to control and experimental groups. Participants in the experimental group use home-based VRT to do rehabilitative exercises for standing balance, stepping, reaching, strengthening and gentle aerobic fitness. Control group participants use an iPad with apps selected to rehabilitate cognition, hand fine motor skills and visual tracking/scanning. Both groups are instructed to perform 30 min of exercise 5 days a week for 6 weeks. VRT intensity and difficulty are monitored and adjusted remotely. Weekly telephone contact is made with all participants. Ability to recruit participants, ability to handle the technology and learn the activities, compliance, safety, enjoyment, perceived efficacy and cost of program delivery will be assessed. A battery of assessments of standing balance, gait and community integration will be assessed for feasibility of completion within this population and potential for improvement following the intervention. Effect sizes will be calculated.

**Discussion:**

The results of this study will be used to support the creation of a definitive randomized controlled trial on the efficacy of home-based VRT for rehabilitation post stroke.

**Trial Registration:**

ClinicalTrials.gov, NCT03261713. Registered on 21 August 2017. Registration amended on 1 June 2018 to decrease enrollment from 40 to 20 due to a cut in study funding and difficulty recruiting participants.

**Electronic supplementary material:**

The online version of this article (10.1186/s13063-019-3438-9) contains supplementary material, which is available to authorized users.

## Introduction and objectives

Stroke causes approximately 17,600 hospital admissions per year in Ontario and 50% of individuals who have had a stroke are left with moderate to severe impairment [1, 2]. Most patients who are discharged from inpatient stroke rehabilitation are only 8–10 weeks post stroke and have not completely recovered. Their central nervous systems are still in a period of enhanced neuroplasticity, during which great functional change can be made [3, 4]. Therapy outcomes are dose-dependent; intensive, high-repetition, task-oriented and task-specific therapies are most effective [5, 6]. Therefore, for the greatest recovery possible, these patients require ongoing, intensive therapy. Most are offered this on an outpatient basis. However, for many reasons (transportation difficulties, distance from the rehabilitation center, weather etc.), not all eligible patients are able to attend outpatient therapy. Also, there is a waiting list and a limited number of outpatient therapy sessions are offered to each patient. Home-based therapy may fill an important role towards increasing the availability of rehabilitation, enabling patients to enhance or prolong their therapy and potentially improving outcomes.

Non-immersive virtual reality training (VRT) uses computer software to track the user’s movements and allow him or her to interact with a game or activity presented on a TV screen. It is convenient, timely, enjoyable and may be used for an unlimited period post stroke [7, 8]. VRT has been shown to benefit upper extremity function, standing balance, gait and overall function in the sub-acute and chronic phases post stroke, at least as much as or more than conventional therapy [7, 9–13].

Home-based VRT offers a promising addition or alternative to existing rehabilitation programs that could make a significant difference in the lives of stroke survivors. A few preliminary studies have investigated the use of home-based VRT for standing balance and upper extremity recovery after stroke and shown potential feasibility of these systems for ongoing rehabilitation in the home [14–18]. Some VRT platforms allow the user to interface via tactile devices (for example, a dynamic standing frame [14] or robotic glove [18]) while others use motion-tracking via a camera [16]. Some platforms use asynchronous monitoring to allow the therapist to monitor VRT usage and performance after the actual event [16] while others use synchronous monitoring to enable the therapist to watch in while the participant exercises; some even require constant real-time patient/therapist interaction [17, 19] throughout the therapy session. Users report high satisfaction with home-based VRT [16, 17], although actual usage can vary greatly [18]. Barriers to the use of home-based VRT include technical issues and lack of previous technical experience [18]. While some previous experience with computers is helpful, those who play video games regularly can become bored with VRT. Facilitators include the flexibility of home-based exercise, support from family and motivation from the VRT itself. Early results, available from a single randomized controlled trial (RCT) with 30 participants, suggest that home-based VRT improves standing balance and gait equally to in-clinic VRT, but that the costs are 44% lower [16].

We wish to add to these early studies of home-based VRT using a virtual reality system (Jintronix Inc.) that was initially developed for stroke rehabilitation and has also been used extensively with healthy and frail elderly individuals. The Jintronix system is marketed for institutional and home use and has a simple-to-use interface, but its home use has not yet been fully evaluated. The games are designed to incorporate motor learning principles such as multiple forms of feedback and task-specific practice that can be progressed to maintain an appropriate level of challenge. Unlike systems used in previous research, the Jintronix system includes a wide selection of games and exercises designed for the rehabilitation of sitting and standing balance, gait and upper extremity use. The system is simple to use and relatively inexpensive; a motion-tracking camera and software eliminates the need for gloves/controllers etc. It is straightforward enough for the patient to use independently; asynchronous monitoring is used to track usage and the therapist can change games and parameters remotely. The purpose of this study is to investigate the feasibility, acceptance and safety of this new, simple-to-use VRT system for use in the home, combined with asynchronous, remote support for the user. The results of this trial will support a definitive RCT in the future.

The primary objective is to assess the feasibility of using VRT in the home with patients post stroke, using quantitative and qualitative methods. Specific objectives are:

1. To estimate the recruitment rate of participants into the study;
2. To assess the ability and compliance of the participants with respect to the components of the research protocol (ability to learn VRT through the training program; ability to comply with the exercise protocol; participant retention);
3. To determine the safety of home-based VRT (presence of minor and major adverse events);
4. To assess the ability of stroke survivors and their study partners to use VRT technology in the home (i.e. technical difficulties, difficulty learning the games);
5. To assess the acceptability of the VRT intervention (enjoyment; perceived efficacy);
6. To estimate the cost for a future definitive RCT on in-home VRT.

The secondary objective is to assess the feasibility of the outcome measures, using quantitative and qualitative methods. Specific objectives are:

1. To assess the feasibility and acceptance of a battery of outcome measures, including physical assessments, questionnaires, an interview and a log book;
2. To assess the potential that home-based VRT might maintain or improve physical outcomes of standing balance, gait and general function and community integration after discharge from hospital-based stroke rehabilitation, compared to those who perform a program of iPad apps designed for fine hand motor skills and cognitive training;
3. To determine the sample size required for a future definitive RCT on in-home VRT.

This study is a prospective, single-site, single-blinded, parallel-group (1:1 ratio) randomized, superiority feasibility trial on the use of VRT for ongoing stroke rehabilitation after discharge from inpatient or outpatient stroke rehabilitation. A feasibility RCT was chosen in order to provide the most useful results to prepare for a future definitive RCT on the efficacy of home-based VRT. iPad apps were chosen as a comparator to VRT because they provide a control group that has equal contact with the researchers and equal time spent in an engaging activity. The use of an active control group (rather than providing control group participants with nothing) was also chosen to facilitate recruitment. The iPad apps chosen to work on hand fine motor control and cognition were not deemed to have any influence on the physical outcome measures of standing balance, gait and gross motor function. The Standard Protocol Items: Recommendation for Interventional Trials (SPIRIT) checklist is available as Additional file 1: Figure S1.

## Methods

### Ethics, consent and permissions

This research is being performed in accordance with the Declaration of Helsinki. Approval was obtained from the Élisabeth Bruyère Research Institute (M16-17-013) and University of Ottawa (A01-15-03) (Canada) Research Ethics Boards. Potential participants are informed of study details, including procedures, risks and benefits, confidentiality and the voluntary nature of participation, before signing the consent form.

### Participants

Potential participants have been recruited from the inpatient and outpatient stroke rehabilitation programs at Élisabeth Bruyère Hospital in Ottawa, ON, Canada, since May 2017. Élisabeth Bruyère Hospital is the largest provider of rehabilitation services for stroke in the Champlain Local Health Integration Network in Eastern Ontario and admits approximately 250 stroke survivors to its inpatient program and 250–300 to its outpatient program each year.

Stroke survivors are eligible for the trial if they (1) have had a stroke (ischemic or hemorrhagic) resulting in physical impairment; (2) have sufficient preserved cognitive ability to learn VRT; (3) are receiving inpatient or outpatient stroke rehabilitation services; (4) are able to stand independently for at least 2 min; (5) have a study partner who can attend two training sessions with the participant and is able to be in the home when the participant is doing VRT; (6) can read, speak and understand English; (7) live within 50 km of Élisabeth Bruyère Hospital; (8) are able and willing to attend four appointments at Élisabeth Bruyère Hospital (two for assessment, two for training); (9) will not be travelling away from home for more than 2 days a week for the duration of the study and (10) have sufficient space in their home to do VRT safely. Patients are excluded if they have an unstable medical condition, seizures or vertigo, or are unable to safely perform mild to moderate exercise. Participation in other, non-VRT exercise or rehabilitation programs post discharge does not influence their eligibility in this feasibility trial. The presence of expressive aphasia is not a definitive exclusion criterion.

Members of a patient’s circle of care (physicians, residents, nurses, physiotherapists, occupational therapists, outpatient program triage nurse and social workers or their students) screen patients for the study using the aforementioned criteria. If the criteria are met, the member of the patient’s circle of care asks the patient if he or she would like to hear about a study using video games and exercise at home. If the patient consents verbally, their name is identified to the research associate (RA), who approaches the patient and provides full details on the study. Interested patients are given an opportunity to ask questions before written informed consent is obtained.

### Sample size and recruitment

No sample size calculation was performed as this is a feasibility trial. The sample size of two groups of 20 is based on available time and recruitment expectations and funding level. It is expected that having 20 participants in the VRT arm of the study will be enough to discern the feasibility of home-based VRT. It is deemed reasonable that 40 out of the estimated 375 stroke rehabilitation inpatients and the 375–450 patients who attend outpatient stroke rehabilitation over 18 months will be eligible and interested in the study. Members of the clinical care team will be made aware of the study through their clinical manager and attendance at team meetings. The study will be stopped prematurely if serious adverse effects occur.

### Randomization, allocation and blinding

Participants are enrolled by the RA. A computer-based randomization system (Sealed Envelope, London, UK) is used to allocate participants in a 1:1 ratio to either experimental or control groups, using permuted blocks while maintaining concealment. The research physiotherapist (PT) accesses the system immediately after the participant is enrolled; the allocation is provided immediately. The only person blinded to treatment allocation is the RA, who performs the outcome measures. The research PT is blinded to the results of the outcome measures. If the participant is undergoing rehabilitation during the study period (for example, outpatient or community-based rehabilitation), the rehabilitation professional is informed of their patient’s participation in the study, to ensure that the home-based exercise is compatible with the participant’s therapy. The PT will reveal a participant’s allocation in the case of serious adverse effects.

### Interventions

**Fig. 1:**
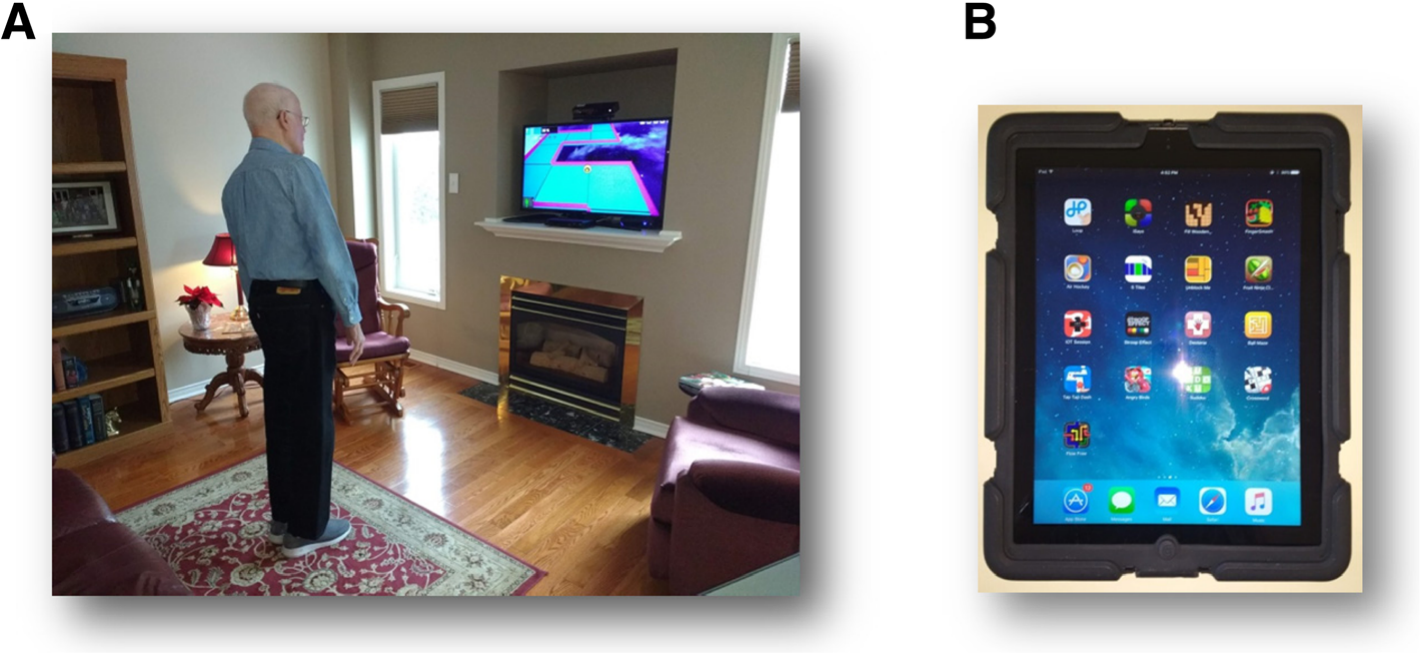
**a** Experimental intervention – home-based virtual reality training targeting standing balance, stepping, reaching, strengthening and aerobic exercise. **b** Control intervention – iPad apps targeting cognition and hand fine motor control

VRT in the experimental group is provided using Jintronix Rehabilitation software (Jintronix, Montreal, Canada). A Kinect camera (Microsoft Canada Co., Mississauga, Canada) captures the movements of the participant using infrared technology and allows him or her to control an avatar, which interacts with an activity presented on a TV screen (Fig. 1a). Several games and activities are available to train participants in standing balance (e.g. moving a ball along a maze, slalom skiing), reaching (e.g. putting dishes away), stepping (e.g. stepping onto tablets placed in a circle, whack-a-mole), gentle strengthening (e.g. standing hip abduction, arm circles, sit-to-stand) and aerobic exercises (e.g. marching on the spot). Game and activity difficulty can be increased by requiring more repetitions or greater speed, distance and/or accuracy. Specific games and activities, and their parameters are selected for each participant based on his or her physical abilities, fall risk, rehabilitation goals and tolerance.

Participants in the control group are provided with an iPad (Apple Canada, Toronto, ON), which contains a selection of apps suited for memory and cognition (e.g. Unblock Me, Fill Wooden Blocks, iSays, Stroop Effect, iOT session, Sudoku, crossword), visual scanning and tracking (e.g. iOT session), and fine motor skills (e.g. writing practice, iOT session, 5 Tiles, Dexteria, Tap Tap Dash, Ball Maze, Fruit Ninja, Finger Smash) (Fig. 1b).

### Outcome measures

To address each primary objective as numbered, the following outcome measures are used:

1. The number of participants recruited over 18 months will be determined relative to the number who met the study participation criteria and the number who were approached by the clinical staff and the RA. The inability to recruit at least 30 participants will require the researchers to alter recruitment procedures for a definitive RCT.
2. Ability to comply with the research protocol will be assessed through notes taken by the research PT and comments provided by the participant and study partner in the log book, on the phone and at the interview. If a majority of participants perform less than 450 min of VRT over the 6 weeks (900 min is the requirement of the protocol), the use of VRT as a motivation to encourage people to exercise post stroke will be reconsidered and reasons why will be discerned. Retention of participants will be measured and a loss of more than 25% will suggest that changes should be made to the protocol.
3. The presence of adverse effects will be recorded from telephone conversations with the participant and through the log book. The occurrence of major adverse effects (e.g. falls with serious injuries) would suggest that home-based VRT, as used in this protocol, is too risky to continue.
4. Ability to use VRT in the home will also be assessed through comments written in the log book or made by the participants during VRT installation and follow-up phone calls and in the interview. Use and progression of the VRT program will be monitored asynchronously by the research PT. Poor learning would be implicated if no progression is observed. Also, expressions of frustration or confusion with the games will indicate poor ability to do VRT.
5. Acceptability of VRT will also be assessed using information gleaned from the log books and follow-up phone calls and from the Physical Activity Enjoyment Scale, which rates (from 1 to 7) 18 statements of one’s feelings about physical activity (PACES) [20], administered at the post hoc assessment only.
6. Costs related to the VRT equipment/licences, travel and salaries will be calculated, in order to prepare a budget for a definitive RCT.

The following outcomes will be used to address each secondary objective.

1. The first secondary objective (feasibility and acceptance of a battery of outcome measures) will be assessed by recording the completion of 11 outcome measures, including physical outcomes (5 tests), questionnaires (4 in total), a log book and a semi-structured interview. The inability of all participants to complete the outcome measures, within a reasonable time frame (2 h) will result in re-thinking the use of each measure in a definitive RCT.
2. The potential for VRT to maintain or improve standing balance, gait, general function and community integration post stroke will be assessed with the following tests:
  - Berg Balance Scale (BBS) [21]. The BBS tests balance and mobility using 14 items scored from 0 to 4.
  - Timed Up And Go (TUG) [22–24]. The TUG assesses the time required to stand up, walk 3 m, return and sit down. There are 3 versions, original, manual (performed while holding a cup of water) and cognitive (performed while also doing a cognitive task).
  - Five Times Sit-To-Stand Test (FTSST) [25]. The FTSST tests the time it takes to stand up from a chair and sit down again five times.
  - Community Balance and Mobility Scale (CB&M) [26]. The CB&M assesses more difficult balance tasks that are relevant to community ambulation.
  - Quantitative analysis of quiet stance and limits of stability in standing [27]. These tests provide quantitative information about the neural mechanisms of postural control [1].
  - Stroke Impact Scale (SIS) [28]. The SIS assesses health status after stroke.
  - Reintegration to Normal Living Index (RNLI) [29]. The RNLI assesses the degree to which individuals have reintegrated back into normal social activities after illness. It has acceptable reliability and validity.

All of these scales have adequate to excellent reliability, validity and responsiveness to change (except responsiveness to change for the FTSST) [26, 28, 30–36].The Motivation for Physical Activity Questionnaire will be administered before the VRT protocol only, to better describe the participant sample [37].

3. The results of the BBS will be assessed and used to calculate an effect size in order to estimate the sample size for a definitive RCT.

### Procedures and participant timeline

**Fig. 2:**
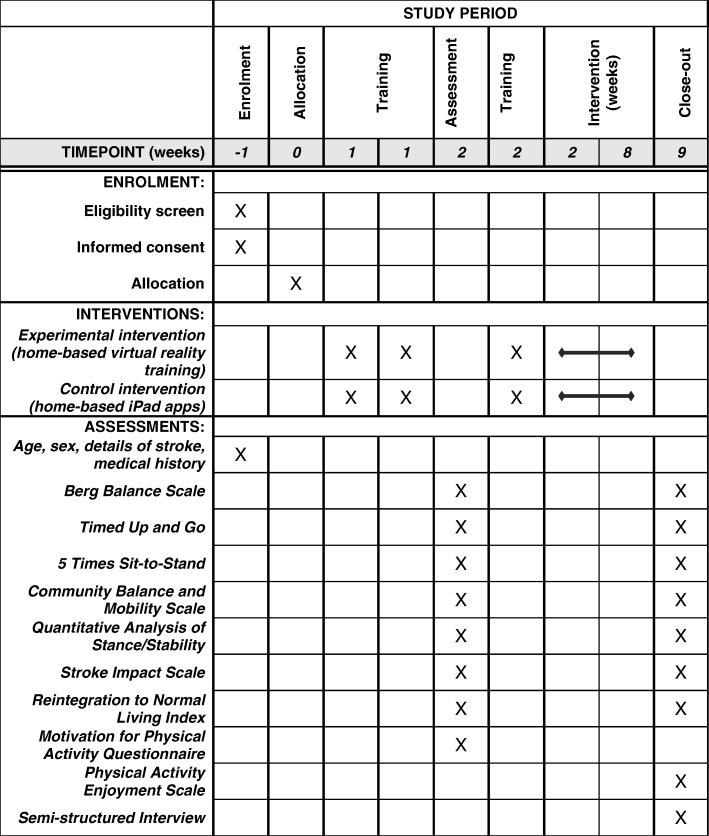
Schedule of enrolment, interventions, and assessments

Participants in both groups (and their study partners) attend a total of 4 sessions at Élisabeth Bruyère Hospital (see Fig. 2). The first 3 sessions occur a week or two before discharge from inpatient or outpatient rehabilitation. At sessions 1 and 2, each lasting 45–60 min, participants and their study partners are trained on how to use the VRT system or iPad and how to play the games. They are also instructed what to do if something goes wrong (for example, if the participant falls or the equipment did not work). Participants are given an instruction manual. Pre-outcome measures are performed by the RA at a third session, lasting approximately 1½ h.

After assessment, the research PT visits the participant’s home to install the equipment and finish training. The games, safety considerations and follow-up procedures are reviewed with the participant and his or her study partner. Participants in both groups are instructed to perform their exercises five times a week for 6 weeks. VRT sessions are designed to run for approximately 30 min of activity time and participants in the iPad group are asked to use it for at least 30 min a day. This amount of additional training (15 h) has been shown to produce a significant improvement in activities of daily living post stroke [38]. The study partner must be in the home of the participant while he or she is doing VRT.

All participants are contacted by the research PT via telephone or email twice a week for the first week and at least once a week for the following 5 weeks to offer encouragement, suggest modifications to the games and identify any safety issues or technical problems. Participants are also invited to contact the research PT as needed. For those in the experimental group, the research PT monitors the compliance and success rate of VRT at least once a week using the remote access feature of the VRT system, and can modify the games if necessary.

Participants in both groups are provided with a logbook in which to record technical issues with the VR equipment, thoughts on how the games are going and if progression was required, safety concerns and adverse events and changes to the exercise environment. Those in the control group must also record the time spent on the iPad apps each day and which apps are used. Participants are encouraged to do as many activities of daily living (ADL) and instrumental ADLs as they wish, including walking, participation in exercise groups and therapy. Throughout training and during the intervention, every attempt is made to equalize contact time with the research personnel between the two groups.

After the 6-week exercise protocol is completed, participants return to Élisabeth Bruyère Hospital for their post-intervention assessment (1½ h). During the post-intervention assessment, the physical outcome measures and community integration questionnaires are repeated, the PACES is administered and a semi-structured interview takes place with a second RA in which participants and their study partners are asked several questions about their experience with VRT (Additional file 2: Figure S2). Once outcome measures are completed the research PT visits the participant’s home for a second time to remove the equipment.

### Data management and analysis

All data will be coded with a participant number; the code breaker will be kept in a different area on the computer server in a password-protected file. A database will be kept with demographic information (age, sex, details of stroke, medical history etc.) and the results of the physical outcome measures and questionnaires. Data on recruitment, retention, number of exercise sessions undertaken in 6 weeks and the number of minutes for each session will be included. Major and minor adverse effects will be recorded and ancillary care will be provided if required.

The pre-intervention and post-intervention scores for the BBS, TUG, FTSST, CB&M, SIS, RNLI and PACES will be reported as mean (95% confidence intervals). Missing data will be handled using complete case analysis. Each scale (except the PACES, which is assessed post-intervention only) will be compared using mixed-methods analysis of variance (ANOVA) (within-group factor, time; between-group factor, group; interaction, time x group). For quiet stance and limits of stability in sitting and standing, an ellipse encompassing 95% of the center of pressure data points will be computed. The area, anteroposterior and mediolateral dimensions of this ellipse will be determined. Results will be compared over time using mixed-methods ANOVA as aforementioned. Linear regression will be used to analyze what proportion of the results of the physical outcome measures is explained by motivation to undertake physical activity (assessed with the Motivation for Physical Activity Questionnaire and the PACES).

Recordings from the interviews will be transcribed. Qualitative data from the transcribed interviews, log books and records of contacts with the participants will be grouped, organized and classified into categories or themes [39, 40]. The categories will be examined to answer primary research objectives 2, 3, 4 and 5, and secondary objective 1 on the enjoyment of VRT, safety, difficulties with the technology or exercises and perceived effectiveness. The datasets created during this study will be available from the corresponding author on reasonable request.

## Discussion

The provision of stroke care in the home or community, according to the needs of the patient and family, is advocated by best practice [2]. VRT is a novel modality with which to provide home-based rehabilitative exercise. Newer VRT systems are small, easy to use and can be monitored by a clinician asynchronously, making them ideal to be used in the home. Home-based VRT is appropriate in several circumstances. Individuals with mild stroke, who are discharged home from acute care, can use it to enhance their recovery back to normal function. It is also beneficial for those with more severe strokes, to maintain or enhance treatment intensity after discharge from inpatient rehabilitation. VRT can be considered a supplemental therapy to traditional outpatient or community-based rehabilitation, which is typically attended one to three times a week. It can also allow for the continuation of therapeutic exercise once a patient is discharged from formal rehabilitation. An increase of 15 h or more of rehabilitative exercise is required to increase function after stroke [38]. This amount of work may be easier to achieve if patients are able to do exercise at home, daily, on their own schedule.

Because of the potential for increased intensity, home-based VRT may enhance recovery from stroke and improve function. This feasibility RCT is the first step in testing that hypothesis. So far, there has only been one small RCT on home-based VRT, which used a single Kinect-based VRT activity at home three times a week along with clinic visits twice a week [16]. Our study uses a VRT system that provides a greater selection of activities (*n* = ~ 29) and exercises (*n* = ~ 55), which provides much greater customization to a patients’ treatment goals. As well, the VRT intervention in our protocol is solely home-based.

We expect that VRT will be deemed to be feasible, in that the VRT equipment will be able to be installed in participants’ homes and that they will be able to successfully use the technology to learn and progress the VRT games and activities. We also expect that participants will enjoy VRT and will perceive that it helps them in their recovery. Finally, we expect that VRT will not cause any adverse effects, such as falls or other injuries.

The results of this study will be submitted as a manuscript to a relevant journal and presented at the 2019 Canadian Stroke Congress and also at smaller, local meetings and rounds. Wider dissemination of the research to the stroke research community, clinicians and the public will be provided by the Canadian Partnership for Stroke Recovery and also by Bruyère Continuing Care, along with its collaboration with local news organizations.

One limitation of this feasibility RCT is its small sample size. While it is appropriate to do a feasibility study before time and funds are committed for a definitive RCT, this will limit its ability to detect significant differences between groups.

Looking ahead from this feasibility study, we will plan a definitive study to test the efficacy of home-based VRT in improving physical outcomes (standing balance, gait and overall function). The preliminary results will inform the future RCT about such parameters as sample size, control intervention, primary and secondary outcome measures, number of weeks of VRT, length of VRT sessions and number per week. Practical information such as recruitment rate and costs will also be important to inform a future proposal. We are currently engaged in knowledge translation research teaching of occupational therapists, physiotherapists, rehabilitation assistants and recreation therapists to use VRT in the clinic and have plans to expand further into this area. In the future we hope to add the home-based component to the clinician training.

There is an escalating trend toward increasing the use of technology in the home. Home-based VRT is poised to follow this trend, with the expectation that it will increase rehabilitation intensity and lead to improved functional outcomes after stroke.

## Additional files

Additional file 1: **Figure S1.** Completed SPIRIT 2013 Checklist. (DOC 122 kb)

Additional file 2: **Figure S2.** Questions for semi-structured interview of participant and study partner, to examine their thoughts on home-based virtual reality for stroke recovery. (DOCX 18 kb)

## Data Availability

The statement, “The datasets created during this study will be available from the corresponding author on reasonable request”, has been included in the “Methods” section of the manuscript.

## Abbreviations

ADL: Activities of daily living
ANOVA: Analysis of variance
BBS: Berg Balance Scale
CB&M: Community Balance and Mobility Scale
FTSST: Five Times Sit-to-Stand Test
PACES: Physical Activity Enjoyment Scale
PT: Research physiotherapist
RA: Research associate
RCT: Randomized controlled trial
RNLI: Reintegration to Normal Living Index
SIS: Stroke Impact Scale
TUG: Timed Up and Go
VRT: Virtual reality training
