# Supplementary material for: Home-based virtual reality training after discharge from hospital-based stroke rehabilitation: a parallel randomized feasibility trial
Source: Trials. 2019 Jun 7;20:333. doi: 10.1186/s13063-019-3438-9 (PMC6555916; doi:10.1186/s13063-019-3438-9)
Supplement: Supplementary file 2 — Figure S2. Questions for semi-structured interview of participant and study partner, to examine their thoughts on home-based virtual reality for stroke recovery. (DOCX 18 kb) [file 13063_2019_3438_MOESM2_ESM.docx]

**Home-based virtual reality training after discharge from hospital-based stroke rehabilitation: A feasibility study.**

Thank you for taking the time to answer some questions about your experience with the virtual reality system.

1. You used the system (insert #) times.
2. What has been your experience using the virtual reality system? [*Let the person respond. Then probe…]*

What aspects of the experience were positive?

What aspects of the experience were negative?

1. Were any exercises or games that you did with the virtual reality system particularly useful? [*If necessary, probe…*]

Why was this?

1. Do you think that using the virtual reality system helped your recovery? [*If necessary, probe…*]

Can you give some examples why or why not?

1. Do you think that it was a good idea to spend time doing virtual reality training rather than other activities? [*Let the person respond. Then probe…]*
2. Do you have any suggestions for how we could improve the use of virtual reality system as a part of a home exercise program after stroke?
3. Would you be interested in continuing to use it at home after this study is over?

What would help you to continue to use it at home?

What would be a challenge if you wanted to continue using it at home?

**Figure S2**: Questions for semi-structured interview of participant and study partner, to examine their thoughts on home-based virtual reality for stroke recovery.
